# Fracture of the frontal sinus: complexity of multidisciplinary treatment

**DOI:** 10.3389/fsurg.2025.1588864

**Published:** 2026-01-06

**Authors:** Vinicius Arruda Vasconcelos, Lucas Cavalieri Pereira, Ana Julia Coral, Laura Diaz, Bruno Nifossi Prado

**Affiliations:** 1 Department of Oral and Maxillofacial Surgery, Piracicaba, Brazil; 2 Department of Oral and Maxillofacial Surgery, HFC Healthy Hospital and Unimed Hospital, São Leopoldo Mandic University, Piracicaba, Brazil; 3 CROM – Centro De Reabilitação Oral E Maxilofacial, HFC Healthy Hospital, Piracicaba, Brazil; 4 Department of Oral and Maxillofacial Surgery, HFC Healthy Hospital, São Leopoldo Mandic University, Piracicaba, Brazil; 5 Departament of Oral and Maxillofacial Surgery, São Leopoldo Mandic University, Piracicaba, Brazil

**Keywords:** frontal sinus fracture, maxillofacial trauma, cranialization, nasofrontal duct, multidisciplinary management

## Abstract

**Objective:**

This study aims to retrospectively analyze the management of frontal sinus fractures by a single Oral and Maxillofacial Surgery (OMFS) team over an 8-year period, underscoring epidemiological characteristics, treatment strategies, and clinical outcomes.

**Methods:**

Medical records of patients diagnosed with and treated for frontal sinus fractures between 2017 and 2024 at Piracicaba, São Paulo, Brazil, were reviewed. Data collection included demographic information, etiology, associated injuries, treatment modality, operative time, and complications. All patients underwent computed tomography (CT) evaluation, and treatment decisions followed a standardized institutional algorithm.

**Results:**

A total of 40 patients (39 male, 1 female; mean age 42.9 years) were included. Traffic accidents were the most common cause (37.5%) of injury. Twenty-two patients (55%) were managed conservatively, while 18 (45%) underwent surgery. The mean operative time was 3 h, with a mean interval of 5 days between trauma and surgery. No intraoperative or postoperative complications were reported. The average hospital stay ranged from 3 to 7 days, depending on the presence of associated injuries.

**Conclusions:**

Frontal sinus fractures are challenging injuries that require careful assessment of displacement, nasofrontal duct patency, and patient condition to determine the optimal management approach. Conservative treatment proved effective for non-displaced fractures with a patent nasofrontal duct, whereas surgical intervention was required for displaced or comminuted fractures and provided satisfactory outcomes with minimal morbidity. These findings reinforce the importance of standardized decision-making protocols and multidisciplinary collaboration in optimizing patient prognosis.

## Introduction

Facial trauma is a frequent occurrence in large urban centers due to heavy traffic and high rates of interpersonal violence. Frontal sinus fractures account for approximately 5%–15% of all facial fractures (1–4). The frontal bone is one of the strongest bones in the craniofacial skeleton, fracturing only under high-energy trauma (800–2,200 pounds of force) (3).

For frontal sinus fractures to occur, a high-intensity trauma directly to the region is necessary. Due to the intensity of the trauma in the area, frontal sinus fractures are often accompanied by traumatic brain injury (TBI), intracranial lesions, orbital injuries, or multiple facial fractures or injuries in other regions of the body, typically requiring multidisciplinary assessment and treatment (5, 6). Frontal sinus fractures can involve the anterior wall of the sinus, the posterior wall, and the frontal sinus duct and are described as non-displaced, displaced, or comminuted (4).

During physical examination of each patient, signs such as depression of the frontal region, lacerations in the tissue, edema, and bruising were noted as indications of the presence of fractures in the frontal sinus area (7). Fractures of the orbital roof may also be associated with superior orbital fissure syndrome or apex orbital syndrome, thus compromising the eye movement of the patient (7). Another important manifestation is the nasal drainage of cerebrospinal fluid, which indicates a fracture of the posterior wall of the frontal sinus and can inform future treatment (4–7).

There are three key considerations in the treatment of frontal sinus fractures: the degree of fracture displacement or comminution, involvement of the anterior and posterior walls of the frontal sinus, and the patency of the frontonasal duct (8–10). Treatment can be conservative, involving observation of signs and symptoms over a long period, or surgical, which includes repair of the anterior wall of the frontal sinus, obliteration of the frontonasal duct, and cranialization for repair of the posterior wall of the frontal sinus (1, 8–10). The experience of the surgical team and the use of an algorithm assist in selecting the best treatment for the patient (1, 9).

In conservative cases with minor detachments of up to 2 mm, treatment may involve rest and nasal irrigation with saline solution. Even in the simpler cases, long-term follow-up should always be conducted, as many complications from these fractures can be delayed (3, 7).

For surgical interventions, the coronal approach is most commonly used. This approach allows for wide exposure of the upper third of the face, ensures low morbidity, and offers the necessary aesthetics for treating frontal sinus fractures (10). With this access, the reduction and fixation of fractures are made easier, while allowing concurrent cranial procedures, graft procurement, frontonasal duct obliteration, myofascial flap usage, and access to adjacent fractures of the upper orbit and middle third of the face. Other options such as access through the laceration itself, superciliary access, or the gull-wing approach are utilized for fractures with minimal displacement that require limited fixation (11, 12).

The obliteration of the frontal sinus is deemed necessary in cases of the posterior wall fractures of the frontal sinus that do not require neurosurgical intervention, compromise of the drainage system of the frontal sinus, communications of the anterior wall of the maxillary sinus, chronic infections, and non-malignant conditions (13). Upon obliteration of the frontal sinus, the intracranial contents are isolated, correcting fluid leaks, preventing infections and local sequelae, and restoring functional integrity and the frontal aesthetic contour (3).

To avoid the risk of severe infection (meningitis, encephalitis, brain abscess, frontal sinus abscess, and osteomyelitis), intervention for frontal sinus fractures is recommended within 48 h following trauma. However, clinical conditions may delay surgical management, increasing the risk of infectious complications if not addressed properly (14).

The complications of frontal sinus fractures are of significant concern due to the anatomical region and the close proximity to highly sensitive structures such as the brain and the orbits (15). Major complications can occur in up to 10% of cases and can be classified as early (up to 6 months) or late (after 6 months) (4). Early complications include brain injury, aesthetic deformities related to the contour of the skull, paresthesia of the supraorbital and supratrochlear nerves, meningitis, sinusitis, and cerebrospinal fluid leakage. Late complications include mucocele, mucopyocele, cerebral abscess, meningitis, hypoesthesia, chronic pain, edema, visual disturbances, and contour deformity (4, 7, 14, 15).

Complications may also arise from the surgical access used for the procedure, including scarring, alopecia, deficit of the frontal branch of the facial nerve, and infection (10). The management of these complications can be conservative, utilizing medications or local clinical procedures, until further surgical or neurosurgical intervention is required. Due to these factors, there is a significant need for early diagnosis of complications.

Therefore, the aim of this study was to retrospectively analyze the management of frontal sinus fractures by a single Oral and Maxillofacial Surgery team, with emphasis on the epidemiological profile, treatment strategies, and clinical outcomes, to identify factors that may improve therapeutic decision-making.

## Methods

The study was conducted in accordance with the Declaration of Helsinki and had the ethical approval committee at São Leopoldo Mandic University.

This retrospective study analyzed the medical records of patients with frontal sinus fractures treated between 2017 and 2024 (8 years) in the city of Piracicaba (423,000 inhabitants), São Paulo (Brazil). Cases were drawn from three trauma reference hospitals in the region, and all patients were diagnosed and treated by the same Oral and Maxillofacial Surgery team, following a standardized diagnostic and therapeutic workflow based on Bell's algorithm (1).

All patients underwent computed tomography (CT) scans for diagnostic confirmation, fracture classification, and surgical planning when indicated. The choice between conservative and surgical management was guided by the degree of displacement, integrity of the nasofrontal duct, and involvement of the posterior wall. Conservative treatment was indicated for non-displaced fractures (<2 mm) with a patent nasofrontal duct, while surgical treatment was selected for displaced, comminuted, or duct-obstructed cases.

The necessary inclusion criterion was the complete medical record, from the initial diagnosis to the outcome of treatment and a minimum 6 months of post-surgical follow-up.

Data were tabulated in Microsoft Excel (Microsoft Corporation, Redmond, WA), which was used to generate the tables and graphs necessary for our study.

Patients were categorized according to gender, age, trauma etiology, type of treatment, and associated injuries.

The mean follow-up period for included patients was 6 months or more, conducted through outpatient evaluations and imaging follow-up when necessary, thereby ensuring proper healing and detection of late complications.

Perioperative management followed a standardized protocol: Dexamethasone 8 mg every 8 h for 3 days, Dipyrone every 6 h, Tramadol as needed for pain control, Ondansetron for nausea, and Omeprazole once daily in the morning. In most polytraumatized patients, medication was managed by the intensive care unit team; however, when hospitalized under the maxillofacial surgery service, this regimen was maintained. The preferred antibiotic was Cefazolin 2 g IV or Amoxicillin–Clavulanate when indicated.

Operative time, surgical delay, and blood loss were recorded when possible. The mean operative time was approximately 3 h, and the average interval between injury and surgery was 5 days. No intraoperative or postoperative complications were reported.

## Results

### Demographics and the mechanism of injury

The study reviewed 40 patients (39 male, 1 female) who were treated and followed up for frontal sinus fractures, with an average age of 42.9 years (range 1–89 years). The average follow-up period was 8 years (2017–2024). The mechanisms of injury were as follows: traffic accident in 15 patients, falls in seven patients, physical aggression in five patients, sports accidents in seven patients, and labor accidents in six patients (Table 1).

**Table 1:** Patient demographics and summary of the mechanism of injuries.

Demographics	*n* (%)
Total no. of patients	40 (100)
Average age, years (range)	42.9 (1–89)
Sex, *n*	
Male	39 (97.5)
Female	1 (2.5)
Average follow-up, years (range)	8 years (2017–2024)
Mechanism of injury	
Traffic accident	15 (37.5)
Fall	7 (17.5)
Physical aggression	5 (12.5)
Sports accident	7 (17.5)
Labor accident	6 (15.5)

The mean operative time was 3 h, and the average interval between injury and surgery was 5 days. Estimated blood loss was minimal in all cases, and no intraoperative or postoperative complications were observed. Antibiotic prophylaxis was performed with Cefazolin 2 g IV or Amoxicillin–Clavulanate in accordance with the institutional protocol. The average hospital stay ranged from 3 to 7 days, depending on trauma severity and systemic conditions.

### Associated injuries

The injuries associated with the frontal sinus fractures are summarized in Table 2. The most common fractures were soft tissue lacerations in 12 patients and zygomatic-maxillary complex fractures in eight patients. Other injuries associated with the frontal sinus fractures included nasal bone fracture in four cases, orbital roof fracture in seven cases, orbital floor fracture in five cases, maxillary fractures (Le Fort I, II, III) in eight cases, posterior wall fracture of the frontal sinus in five cases, ethmoid fracture in one case, ocular laceration in two cases, and soft tissue laceration in 12 cases; no cases of mandibular fracture were observed.

**Table 2:** Injuries associated with frontal sinus fractures.

Associated injuries	*n*
Nasal bone	4
Zygomaticomaxillary complex	8
Orbital roof	7
Orbital floor	5
Maxillary fracture (Le fort, I, II, III)	8
Mandible	0
Posterior wall of frontal sinus	5
Ethmoid fracture	1
Eye laceration	2
Soft tissue laceration	12

### Frontal sinus fracture characteristics and management strategy

A total of 22 patients received conservative treatment (55%), while 18 patients were treated surgically. Of the 40 patients, 34 (85%) had isolated anterior table fractures, one patient (2.5%) had an isolated posterior table fracture, and five patients (12.5%) had fractures involving both anterior and posterior tables (Table 3). A total of 23 patients (57.5%) had comminuted frontal sinus fractures and 17 patients (42.5%) had simple fractures. Both fracture patterns were significantly associated with surgical intervention [odds ratio (OR) = 15, *p* < .001]. Anterior or posterior table involvement was not found to be associated with the choice of surgical repair vs. observation (*p* = 0.650).

**Table 3:** Frontal sinus fracture characteristics and management strategy.

	*n* (%)	Conservative treatment	Surgical treatment	Odds ratio	95% CI	*p*-Value
Fracture pattern	40	-	-	15.0	2.73–82.3	>.001
Simple	17 (42.5)	15	2	-	-	
Comminuted	23 (57.5)	7	16	-	-	
Table involved	40	-	-	-	-	0.650
Isolated anterior	34 (85)	18	16	-	-	
Isolated posterior	1 (2.5)	1	0	-	-	
Anterior + posterior	5 (12.5)	3	2	-	-	

In patients treated conservatively, follow-up consisted of clinical and radiographic evaluations at 15, 30, and 90 days, then every 6 months thereafter. None of the patients under observation developed delayed complications or required further intervention. Among the surgical cases, postoperative recovery was uneventful, and all patients maintained adequate aesthetic and functional outcomes throughout follow-up.

### Management strategy of frontal sinus fractures by table involvement

Involvement of the anterior table, posterior table, or both tables was not associated with management strategy, operative approach, or operation type (*p* = 0.456 and *p* = 0.105, respectively) (Table 4). Only the anterior aspect of the sinus was treated in 13 cases, while craniotomy and repair of the anterior wall of the frontal sinus were performed in two cases, and obliteration of the duct with reconstruction of the anterior wall was necessary in three cases. For internal fixation, plates and screws were used in 10 cases. When there was communication or loss of fragments, titanium mesh was required in eight cases to ensure anatomical and aesthetic reconstruction.

**Table 4:** Management strategy of frontal sinus fractures by table involvement.

	Anterior table	Posterior table	Anterior + posterior table	*p*-Value
Operative approach	16	0	2	0.456
Coronal	14	0	2	
Laceration	4	0	0	
Operation	13	0	0	0.105
Cranialization	2	0	0	
Obliteration	0	1	2	
ORIF	10	0	0	
Mesh titanium required	4	0	2	
Observation	18	1	2	

**Figure 1:**
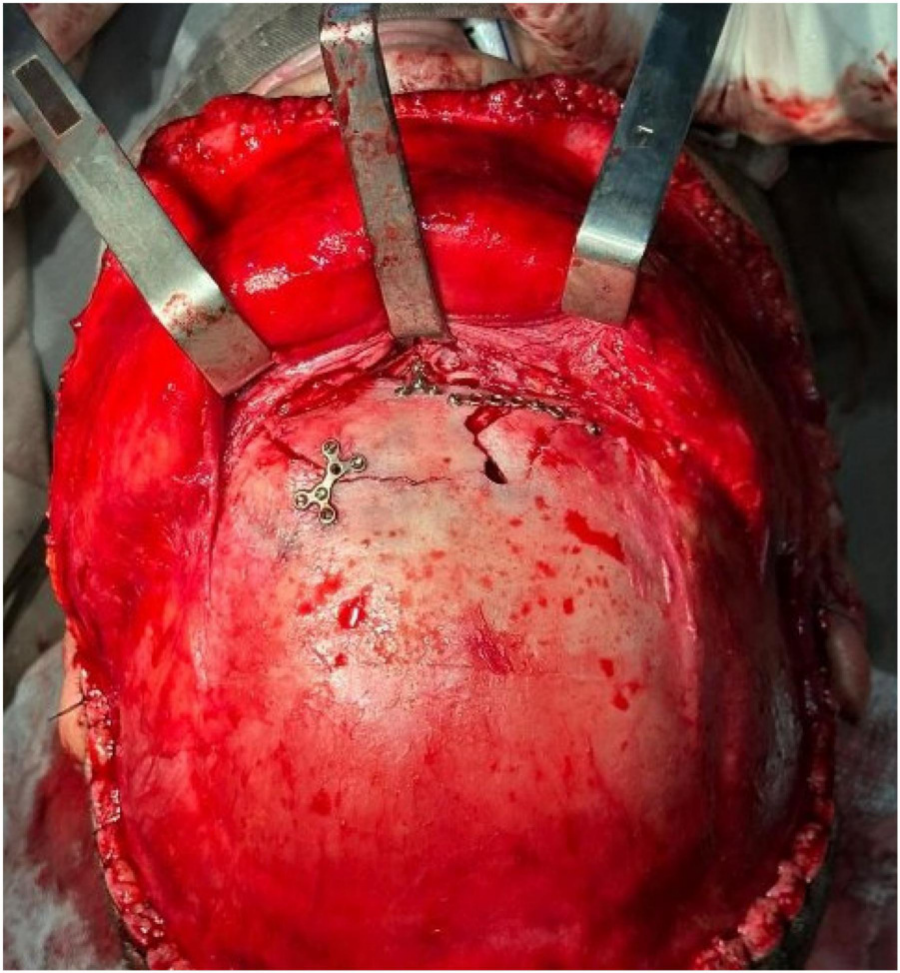
Osteosynthesis of fracture of the anterior wall of the frontal sinus. The image illustrates the surgical technique used to stabilize the defect and recover bone integrity.

**Figure 2:**
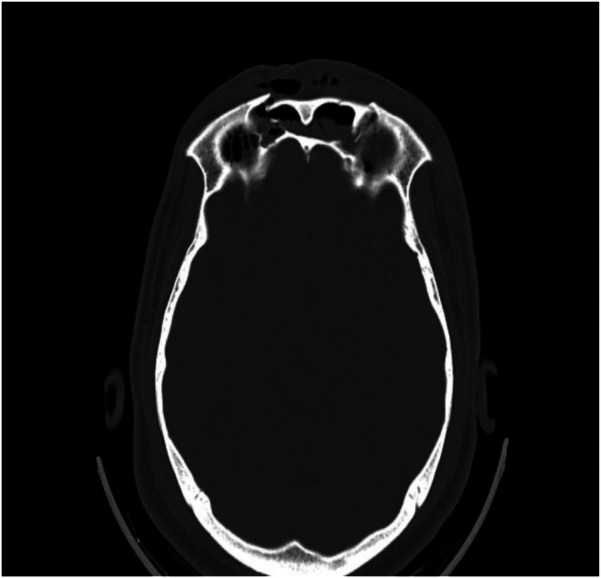
Axial tomography showing the fracture of the anterior wall of the frontal sinus. The image highlights the bone injury and the extent of the fracture.

Representative intraoperative and radiographic images are shown in Figures 1, 2. Figure 1 illustrates the osteosynthesis technique used to stabilize the anterior wall defect, while Figure 2 presents an axial CT scan demonstrating the extent of the anterior wall fracture.

## Discussion

Fractures of the frontal bone require multidisciplinary treatment due to their complexity. In emergency settings, neurosurgery and trauma surgery are typically the first specialties to be called upon in cases of cranial fractures. TBI can lead to significant neurological damage, including functional deficits, hematomas, and intracranial hemorrhages, which necessitate immediate evaluation and management.

Frontal bone fractures, often resulting from high-impact trauma, can lead to significant neurological complications due to their proximity to critical brain structures. These injuries frequently cause concussions, cognitive impairments, brain contusions, and intracranial hematomas, which can result in elevated intracranial pressure and subsequent neurological deficits. Given the complexity and potential for severe outcomes, a multidisciplinary approach is essential for effective management. Collaboration among neurosurgeons, traumatologists, neurologists, intensivists, and rehabilitation specialists is crucial for providing comprehensive care, including surgical intervention, monitoring, and long-term rehabilitation. Early intervention and continuous, specialized care are essential to optimizing patient outcomes and minimizing the risk of permanent neurological damage.

When it comes to fractures of the frontal sinus, there is unanimity among authors regarding the predominance in the male sex (2, 6, 16, 17). Regarding the etiologies of the fractures, our data indicate that traffic accidents are the most prevalent cause (37.5%). Neighboring cities with similar populations (400,000 inhabitants) also exhibited significant incidence rates (2, 16), consistent with findings from the capital city (11 million inhabitants) (17). Only the study by Oslin et al., conducted in a large city with a population of 500,000, identified falls as the most prevalent cause. The age range also varied, with the group aged 20–40 being the most affected (2, 6, 16, 17). In large urban centers, traffic accidents typically increase, primarily due to the large population sample (17).

To reduce accidents, the automotive industry is constantly creating new safety measures for passengers, including more protected cockpits, seat belts, airbags, and new technologies that assist in braking to prevent collisions. These epidemiological findings support the external validity of the present study and reinforce the role of early trauma prevention measures.

In cases involving the posterior wall of the frontal bone, the neurosurgery team must perform cranialization or drainage of intracranial hematoma. In the present series, surgical indications were clearly guided by the degree of displacement and nasofrontal duct patency, indicating that careful preoperative assessment can minimize unnecessary surgical exposure while ensuring proper drainage and sinus function.

The coronal approach is the first choice for the treatment of frontal bone fractures. However, Elkahwagi (18) reported that the coronal approach carries a considerable risk of complications, including a large visible scar, alopecia, and numbness. Therefore, the coronal incision should be reserved for cases of comminuted wide fractures and bilateral fractures. In contrast, an eyebrow incision is preferred (18). However, there is still no consensus on the best treatment for frontal sinus fractures (19).

In the present study, a diagram from the classic work by Bell et al. (1) was utilized to categorize fractures as displaced or non-displaced and assess the integrity of the frontonasal duct. This diagram also evaluates the displacement and comminution of the posterior wall of the frontal sinus, thereby determining the possible treatments: cranialization and repair of the anterior wall, repair of the anterior wall only, obliteration of the frontonasal duct, and repair of the anterior wall. In non-displaced fractures of the frontal sinus with a patent nasofrontal duct, only clinical observation, with head elevation and sinus precaution, is needed.

For frontal sinus fractures with nasofrontal duct outflow obstruction, either sinus obliteration or cranialization is indicated depending on the involvement of the posterior wall. In displaced frontal sinus fractures with no obstruction of the nasofrontal duct, reconstruction of the anterior wall is indicated (7). For cranialization, intracranial hematoma drainage, or repair of the posterior wall fractures, intervention by a neurosurgery team is required. This decision-making process, supported by our data, reinforces the importance of individualized assessment and adherence to well-established surgical algorithms to reduce morbidity and improve outcomes.

A new algorithm proposed by Doonquah et al. (12) is also considered. It includes the possibility of endoscopic treatment approaches for frontal sinus fractures via transnasal access, reducing scars and potential complications. However, the applicability of endoscopic techniques in our cohort was limited due to the small number of suitable cases. Although promising for minor anterior wall fractures, endoscopic approaches should be considered adjunctive rather than primary options, particularly when posterior wall involvement or comminution is present.

There is also the possibility of employing endoscopic approaches, such as brow lifts or hairline techniques, for minor corrections, particularly when there are no fractures of the posterior wall of the frontal sinus or compromise of the frontonasal duct (20). The indication for this technique is patients with isolated anterior table fractures (21). However, the reduction and fixation of fragments (12, 20) imply a significant limitation of this technique. Thus, despite their cosmetic advantage, endoscopic techniques remain restricted to highly selective cases.

Preoperative imaging and informed consent are crucial in planning endoscopic repair for frontal bone fractures. The fracture is exposed and repaired using a Medpor implant, which is stabilized with percutaneous screws. Prefabricated Medpor implants, while offering a better fit, are expensive and take longer to fabricate. This minimally invasive technique ensures stable repair and good cosmetic results; however, it involves increased costs and requires 6 weeks for fabrication of the material (21). The endoscopic approach has also been employed for evaluation of the outflow tract and removal of the obstruction by endoscopic clearance of the frontal recess when required, thereby avoiding another large approach or bone removal that might cause further trauma (18). In summary, while the literature supports endoscopic options for selected cases, open coronal access continues to be the most reliable technique in terms of visibility, safety, and long-term outcomes.

In the present study, the coronal approach was mostly chosen in cases of detachment with a requirement for fixation of the bone fragments. The coronal approach provides extensive surgical access to the upper and middle facial thirds, with minimal long-term complications, excellent aesthetic results, and low morbidity (10). Conservative treatment is recommended for non-displaced fractures with an intact frontonasal duct based on observation and nasal irrigation parameters (1). However, regardless of how straightforward this outcome may be for surgical procedures, long-term follow-up is still recommended in order to prevent late complications such as aesthetic defects and infections, particularly in cases involving fractures of the posterior wall of the frontal sinus (7).

Regarding complications, few events were observed in the present study; however, the follow-up period was 8 years, whereas other studies recommend follow-up periods of 15–20 years (9, 22). Reported rates of complications remain below 10% (2, 4, 6, 7, 16, 17), consistent with our findings. Local hypoesthesia or hyperesthesia is related to the coronal process (10) and manipulation of the supraorbital nerve; it is generally well managed and temporary and is not a plausible patient complaint. This low complication rate highlights the effectiveness of our surgical and postoperative protocols and supports the multidisciplinary integration adopted in our institution.

To reduce the risk factors for complications, it is recommended to operate on fractures within 48 h (14, 22). When possible, drains are to be avoided. Extravasation of cerebrospinal fluid for more than 7 days also increases the risk of complications (22). When considering complications related to frontonasal obliteration, those associated with the graft itself should also be noted, such as necrosis, resorption, infection, and mucosal thickening (13).

Even though craniotomies are rarely analyzed in studies due to the low incidence of surgical requirement on the posterior wall of the frontal sinus, they can produce more severe complications that are difficult to treat clinically or surgically (7). Intravenous antibiotic therapy and new surgical or neurosurgical interventions may be necessary, thus increasing the number of complications and their severity (7, 14, 22). Therefore, surgical planning should always involve interdisciplinary consultation, and patient counseling must emphasize the potential for delayed sequelae even after seemingly successful repair.

## Conclusions

Frontal sinus fractures represent complex injuries that demand accurate diagnosis and coordinated multidisciplinary management involving oral and maxillofacial surgeons, neurosurgeons, and trauma specialists. In this retrospective series, the predominance among male patients was observed, with traffic accidents being the main etiology and an average age of approximately 41 years. Conservative management was the most frequent approach, while surgical treatment was indicated for displaced or comminuted fractures, both resulting in satisfactory functional and aesthetic outcomes with minimal complications. These findings contribute to a better understanding of the epidemiological and clinical aspects of frontal sinus fractures and reinforce the importance of standardized algorithms, early diagnosis, and adequate assessment of fracture displacement and nasofrontal duct integrity to guide treatment planning. Proper indication of conservative or surgical management by trained craniomaxillofacial teams remains essential to optimize prognosis and minimize long-term complications.

## Data Availability

The original contributions presented in the study are included in the article/Supplementary Material, further inquiries can be directed to the corresponding author.
